# Whole Exome Sequencing Reveals Novel PHEX Splice Site Mutations in Patients with Hypophosphatemic Rickets

**DOI:** 10.1371/journal.pone.0130729

**Published:** 2015-06-24

**Authors:** Sara L. Ma, Virginia Vega-Warner, Christopher Gillies, Matthew G. Sampson, Vijay Kher, Sidharth K. Sethi, Edgar A. Otto

**Affiliations:** 1 College of Literature, Science, and the Arts, University of Michigan, Ann Arbor, MI, United States of America; 2 Division of Nephrology, Department of Pediatrics and Communicable Diseases, University of Michigan, Ann Arbor, MI, United States of America; 3 Kidney and Urology Institute, Medanta, The Medicity Hospital, Gurgaon, India; Queen Mary Hospital, HONG KONG

## Abstract

**Objective:**

Hypophosphatemic rickets (HR) is a heterogeneous genetic phosphate wasting disorder. The disease is most commonly caused by mutations in the *PHEX* gene located on the X-chromosome or by mutations in *CLCN5*, *DMP1*, *ENPP1*, *FGF23*, and *SLC34A3*. The aims of this study were to perform molecular diagnostics for four patients with HR of Indian origin (two independent families) and to describe their clinical features.

**Methods:**

We performed whole exome sequencing (WES) for the affected mother of two boys who also displayed the typical features of HR, including bone malformations and phosphate wasting. B-lymphoblast cell lines were established by EBV transformation and subsequent RT-PCR to investigate an uncommon splice site variant found by WES. An *in silico* analysis was done to obtain accurate nucleotide frequency occurrences of consensus splice positions other than the canonical sites of all human exons. Additionally, we applied direct Sanger sequencing for all exons and exon/intron boundaries of the *PHEX* gene for an affected girl from an independent second Indian family.

**Results:**

WES revealed a novel *PHEX* splice acceptor mutation in intron 9 (c.1080-3C>A) in a family with 3 affected individuals with HR. The effect on splicing of this mutation was further investigated by RT-PCR using RNA obtained from a patient’s EBV-transformed lymphoblast cell line. RT-PCR revealed an aberrant splice transcript skipping exons 10-14 which was not observed in control samples, confirming the diagnosis of X-linked dominant hypophosphatemia (XLH). The *in silico* analysis of all human splice sites adjacent to all 327,293 exons across 81,814 transcripts among 20,345 human genes revealed that cytosine is, with 64.3%, the most frequent nucleobase at the minus 3 splice acceptor position, followed by thymidine with 28.7%, adenine with 6.3%, and guanine with 0.8%. We generated frequency tables and pictograms for the extended donor and acceptor splice consensus regions by analyzing all human exons. Direct Sanger sequencing of all *PHEX* exons in a sporadic case with HR from the Indian subcontinent revealed an additional novel *PHEX* mutation (c.1211_1215delACAAAinsTTTACAT, p.Asp404Valfs*5, *de novo*) located in exon 11.

**Conclusions:**

Mutation analyses revealed two novel mutations and helped to confirm the clinical diagnoses of XLH in two families from India. WES helped to analyze all genes implicated in the underlying disease complex. Mutations at splice positions other than the canonical key sites need further functional investigation to support the assertion of pathogenicity.

## Introduction

Hypophosphatemic rickets (HR) is a group of disorders characterized by a defect in renal tubular reabsorption of phosphate, which causes defects in bone mineralization and hypophosphatemia. HR has been divided into four main subtypes: X-linked hypophosphatemic rickets (XLH), autosomal dominant hypophosphatemic rickets, hereditary hypophosphatemic rickets with hypercalciuria, and tumor-induced osteomalacia [1–5]. Accounting for more than 80% of familial HR cases, XLH (MIM 307800) is the most common form of heritable HR, with an occurrence of approximately 1 in 20,000 live births [6–7]. XLH is a dominant disorder with complete penetrance despite varying clinical expressivity [8]. This disease is characterized by massive phosphate wasting, which causes growth retardation, bone malformations, abnormal vitamin D metabolism, and hypophosphatemia [9].

Loss-of-function mutations in a phosphate-regulating gene with homologies to endopeptidases on the X-chromosome (*PHEX)*, have been shown to cause XLH. The gene is composed of 22 exons encoding a protein of 749 amino acids. The protein consists of an intracellular region, a transmembrane domain, and an extracellular domain [10–11]. Inactivating mutations in this gene increase circulating levels of FGF23, a phosphate-regulating hormone, which causes a reduction in renal phosphate reabsorption and abnormal bone mineralization [12]. Though PHEX has a significant role in the phosphate uptake in the renal system, the protein is predominantly expressed in osteoblasts, osteocytes, and odontoblasts rather than in any kidney tissue [13–15].

To date, 365 different *PHEX* mutations have been described in patients with HR, many of which are predicted to lead to protein truncations (58 nonsense mutations, 78 small deletions, 44 small insertions, and 65 splice site mutations; HGMD professional 2014.3 release). Only few novel mutations have been added recently to the public databases [16–18]. Here, we report a familial and a sporadic case with hypophosphatemic rickets, for which genetic mutation analysis revealed a novel splice acceptor site mutation and a novel *de novo* truncating mutation. The novel splice site mutation was further characterized by analyzing aberrant *PHEX* RNA-transcripts detected in patient’s transformed peripheral blood lymphocytes.

## Material and Methods

### Subjects and ethic statement

This study was conducted in collaboration with the Kidney and Urology Institute in Gurgaon, India. Approval for this study and for human subjects research was obtained from the University of Michigan Institutional Review Board (Study ID: HUM00044173) and all subjects provided written informed consent before blood samples, pedigree structure, clinical data and laboratory findings were provided. We investigated four patients from two unrelated families of Indian subcontinent ancestry who were diagnosed with HR based on laboratory indices, clinical indicators, and medical histories. The fractional tubular reabsorption of PO_4_ (TRP) was analyzed based on the standard method and the tubular maximum rate of PO_4_ reabsorption in relation to the glomerular filtration rate (TmPO_4_/GFR) was calculated according to the nomogram of Walton and Bijvoet [19].

### DNA preparation

Genomic DNA was isolated from 5–10 ml peripheral whole blood samples (EDTA) drawn from all affected patients and their parents using the Gentra Puregene Blood kit (Qiagen, Hilden, Germany) according to the manufacturer’s instructions.

### Whole exome sequencing

Exome enrichment was conducted following the manufacturer’s protocol for the ‘NimbleGen SeqCap EZ Human Exome v2.0’ beads (Roche NimbleGen Inc.). The kit interrogates a total of approximately 30,000 genes (~330,000 CCDS exons). Massively parallel sequencing was performed largely as described in Bentley et al. [20]. Whole exome capture and next-generation sequencing was carried out at Otogenetics Ltd. (www.otogenetics.com) on an Illumina HiSeq2000 (Illumina, San Diego, CA) platform and indexed libraries were subjected to paired-end (2×101 bp read length) sequencing-by-synthesis using fluorescent reversible terminators with a blocking group at the 3’-OH group. Three μg DNA of the affected mother E0023-I-2 was submitted for WES. Sequence reads were mapped to the human reference genome assembly (GRCh37/hg19) using CLC Genomics Workbench (version 7.5) software (CLC bio, Aarhus, Denmark). Variants were called, filtered, and prioritized according to their pathogenicity scores (>0.95) obtained from the Polyphen-2 web interface [21], MutationTaster [22], and CADD (>20) [23]. Furthermore, variants were cross-referenced with the Human Gene Mutation Database (HGMD, http://data.mch.mcgill.ca/phexdb), and genes known to be implicated in HR were intensively examined.

### Direct Sanger sequencing of the *PHEX* gene

Primers for PCR amplification of all 22 coding exons and exon/intron boundaries of the *PHEX* gene (NM_000444.4) were designed using the web-based Primer3 (http://biotools.umassmed.edu/bioapps/primer3_www.cgi) software. The sequences are available upon request. A 10 μL PCR reaction was set up with 30 ng genomic DNA, 1.5 pmol of forward and reverse primer each, and 5 μL HotStarTaq Polymerase mixture (Qiagen). DNA amplification was performed on a thermal cycler (Mastercycler; Eppendorf, Hamburg, Germany) and applying a touchdown PCR protocol described earlier [24]. In brief, we applied the following parameters: Initial denaturation at 94°C for 15 min, followed by 24 cycles with an annealing temperature decreasing 0.7°C per cycle, starting at 72°C for 30 sec; denaturation at 94°C for 30 sec, and extension at 72°C for 1 min. An additional 32 cycles were added with 94°C for 30 sec, 55°C for 30 sec, and 72°C for 1 min. The final extension was carried out at 72°C for 10 min. PCR products were diluted 15-fold in pure water and submitted for direct Sanger sequencing on an Applied Biosystems capillary DNA sequencer (Model 3730 XL) without further purification. Sample preparation for Sanger sequencing was done by following the instructions of the BigDye Terminator v3.1 Cycle Sequencing Kit (Applied Biosystems, Foster City, CA).

### TA-cloning of mutant PCR products

In order to obtain clean Sanger sequences of the heterozygous c.1211_1215delACAAAinsTTTACAT (p.Asp404Valfs*5) mutation, detected in female patient E0024-II-1, we cloned the respective 269 and 271 bp long PCR products of PHEX exon 11 using the pCR 2.1-TOPO plasmid vector system (Invitrogen). PCR products generated by using *PHEX* forward primer 5’-TCAGCCATGGGTTTTATCC-3’ and reverse primer 5’-AGGCTGACATTAGCCTGTTG-3’ were ligated into the pCR 2.1-TOPO plasmid vector, subsequently transformed into One Shot chemically competent *E*. *coli* cells applying a 42°C heat shock treatment. Transformed bacteria were incubated for 1 hour in S.O.C. medium (Invitrogen) at 37°C on a shaking incubator (200 rpm) and plated on LB-agar plates supplemented with 50 μg/ml Ampicillin and incubated overnight at 37°C. Plasmid inserts from 7 colonies were sequenced by Sanger using M13 forward and M13 reverse primers. The heterozygous frameshift mutation p.Asp404Valfs*5 was present in 3 out of those 7 clones with 4 clones showing the wild-type sequence.

### Segregation analysis

Following the detection of potential mutations in an affected individual, we performed segregation analysis and applied PCR amplification and direct Sanger sequencing for all family members for the variant(s) in question.

### Epstein-Barr Virus (EBV) immortalization of B-lymphocytes

Peripheral blood mononuclear cells (PBMC) were isolated from 6 ml of peripheral blood on a Ficoll gradient (Ficoll-Paque Plus, GE healthcare) and were suspended in 5 ml of RPMI 1640 medium supplemented with 10% fetal calf serum (Atlanta Biologicals, #S11595), 1x-Glutamax (Gibco), and 7.5 μl Phytohemagglutinin (Gibco, #14175–095). We added 1.5 ml of medium supernatant of the EBV-transformed marmoset B95.8 cell line. About half of the medium was replaced every seven days for four weeks. EBV-transformed B-lymphoblast cells derived from the patients were expanded to about >3 million cells before RNA was extracted. Cells were maintained in complete RPMI1640 medium without penicillin and streptomycin at 37°C and a 5% CO_2_ atmosphere.

### Reverse Transcription Polymerase Chain Reaction (RT-PCR)

We extracted total RNA from EBV-transformed lymphoblast cell lines (3 million cells) from patient E0023-II-2 and from a healthy control individual using Trizol Reagent (Invitrogen) according to the protocol of Chomczyński and Sacchi [25]. First cDNA strand was reverse transcribed using the SuperScript III transcriptase (Invitrogen) together with random hexanucleotide primers. Resulting cDNA was used as template to amplify an 1,636 bp product containing exon 6 to 22 using forward primer PHEX-Ex6-F (5’-GTACAGAAGCCAAGTCTTATCGGGATGC-3’) and reverse primer PHEX-Ex22-R (5’-AATGAAAGTCTCCAGGCCTAAAGCAATG-3’) aligning to the 3’ untranslated region (UTR) of *PHEX*. Aberrantly spliced transcripts were verified following RT-PCR amplification, agarose gel-electrophoretic separation, DNA extraction (QIAquick Gel Extraction Kit, QIAGEN), and subsequent direct Sanger sequencing.

### Genome-wide *in silico* calculation of splice site base-level frequencies

To calculate base-level frequencies of splice donors and acceptors genome-wide, we first downloaded GENCODE v19 and GRCH37.74 from Ensembl (http://www.ensembl.org/index.html). There were a total of 739,829 exon entries in GENCODE v19. After filtering for uniqueness using chromosome, start position and end position, there were 327,293 exons across 81,814 transcripts among 20,345 genes. There were a total of 80,035 transcripts with at least 2 exons. For splice donors, the last exon for each transcript was excluded. There were a total of 257,325 unique donor exons and 69,968 exons (not included in calculation) that were uniquely the last exon for some transcript. We then constructed one query for each of the 257,325 donor exons. We included two bases upstream and twelve bases downstream from the 3' end of each donor exon for a total of fifteen bases. Since many exons have the same 3' position, this resulted in 213,372 unique donor queries. Some sections of the reference sequence where masked out on the Y chromosome, and the total number of queries excluding masked regions was 213,238. For splice acceptors, we excluded the first exon for each transcript, and there were a total of 254,557 unique acceptor exons. There were 72,736 exons (not included in the calculation) that were uniquely the exon first for some transcript. We constructed one query for each of the 254,557 acceptor exons, where we included twenty-three bases upstream and two bases downstream from the 5' end of an acceptor exon for a total of 26 bases. Since many exons have the same start position, some exons were removed, which finally resulted in 206,484 unique acceptor queries. The total number of acceptor queries excluding the masked regions was 206,350. We wrote a custom Perl script to calculate the individual base-level frequencies.

## Results

### Clinical features and laboratory findings

**Fig 1:**
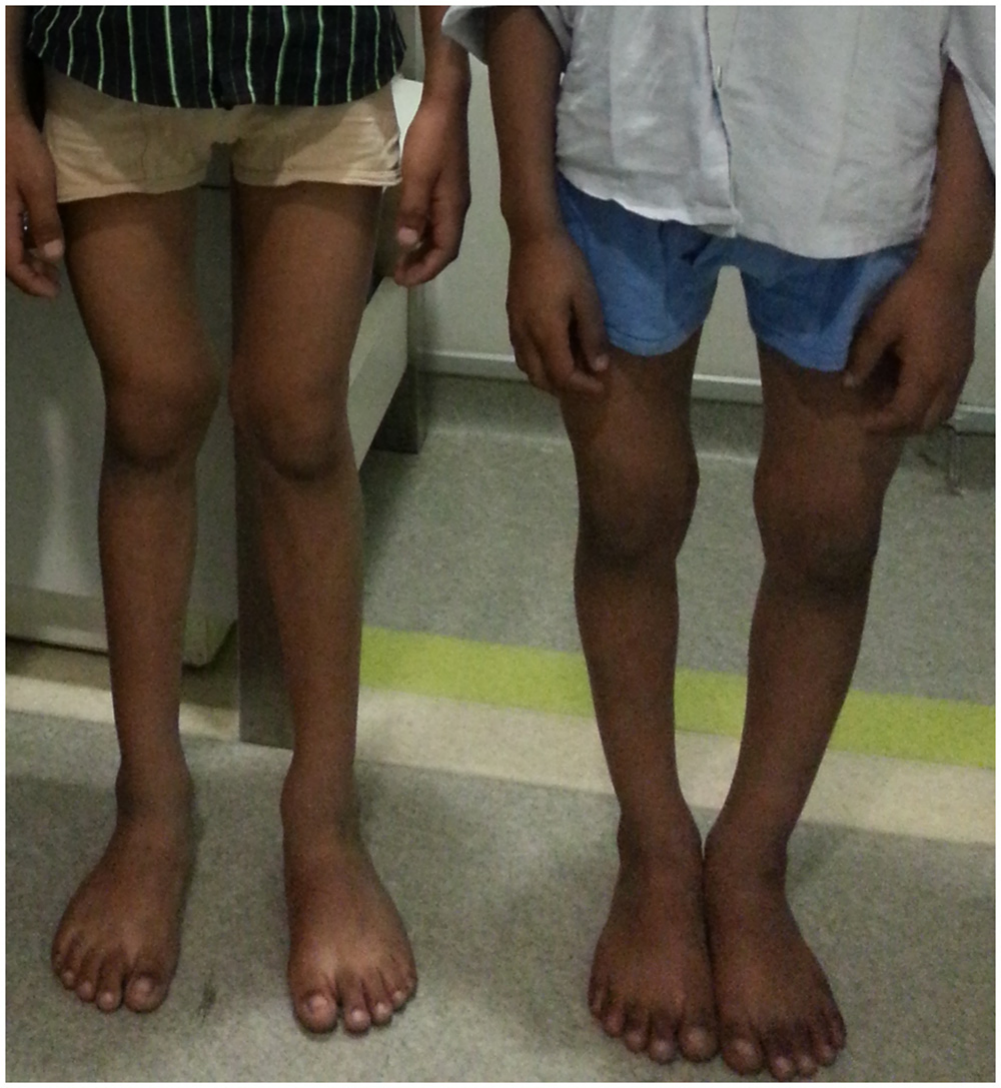
Appearance of two brothers with hypophosphatemic rickets of an Indian family (E0023). Clinical examination of both individuals yielded decreased tubular phosphate reabsorption, growth retardation, and bone malformations typical of hypophosphatemic rickets. Proband II-1 (left) shows shows *genu valgum* “knock-kneed” features while his brother II-2 shows the characteristic signs of *genu varum* “bowlegs”.

Family E0023 from India is a familial case (non-consanguineous) with 3 affected members, the mother and 2 children, diagnosed with hypophosphatemic rickets. The older of the two affected children is a 10 year old boy (II-1) who presented with growth retardation, dental hypoplasia, and *genu valgum* ("knock-knee") deformities of the lower extremities (Fig 1 on the left). His younger brother (II-2) is now 9 years old. He was born at full term and was within average length (51 cm) and weight (3.1 kg) at birth and began walking at the age of 15 months. During the time of examination, he had stunted growth and presented with dental hypoplasia and *genu varum* (“bowlegs”) bone deformities (Fig 1 on the right). Their 33-year-old mother (I-2), upon examination, showed growth retardation, dental hypoplasia, and bony deformations (“knock-knee”), whereas their 37-year-old father (I-1) was healthy.

In family E0024 we investigated a sporadic case with HR, an 8-year-old girl and from India (II-1) who was also born of a non-consanguineous marriage at full term. At delivery, she was within normal length and weight (52 cm, 3.0 kg) and began to walk at the age of 13 months. Her initial clinical examination for bony deformities showed significant growth retardation and lower limb skeletal deformities (Father and mother are both healthy and do not show any signs of HR).

Laboratory tests for all four patients showed low serum phosphorus and serum calcium levels, but decreased tubular reabsorption of PO_4_ (TRP) in relation to the glomerular filtration rate (TmPO_4_/GFR). A compilation of laboratory findings and physical characteristics of all four patients from two independent Indian families are shown in Table 1.

**Table 1:** Clinical data and biochemical results of four patients with hypophosphatemic rickets from two Indian families.

Family	Patient	Gender	Age (yrs)	Height (cm)	Weight (kg)	Clinical Symptoms	Serum P (mg/dL)	Serum Ca (mg/dL)	TRP (%)	TmPO_4_/GFR (mg/dL)
E0023	II-1	Male	10	110	17	Growth retardation, Dental hypoplasia, *Genu valgum* (knock-knee)	2	9.2	34	2.1
E0023	II-2	Male	9	124	23	Growth retardation, Dental hypoplasia, *Genu varum* (bowlegs)	2.9	9.8	30	2.6
E0023	I-2	Female (Mother)	35	135	45	Growth retardation, Dental hypoplasia, *Genu valgum* (knock-knee)	2.2	9.4	24	2.5
E0024	II-1	Female	8	111	18	Growth retardation, *Genu valgum* (knock-knee)	2.6	8.9	38	2.7

All patients received phosphate supplements in form of neutral phosphate (40–60 mg/kg/day) and 1alpha-hydroxyvitamin D_3_ (0.5–1 μg/day). Additionally, all patients were monitored every three months for signs of hypercalciuria or nephrocalcinosis. Laboratory parameters such as serum calcium, serum phosphate, alkaline phosphatase, PTH, and urine calcium/creatinine ratio were also monitored every 3 months, whereas ultrasonography was performed only every 6 months.

### Whole exome sequencing

Aiming to identify the genetic cause underlying the HR disease in the multiplex family E0023 we submitted DNA from the affected mother (E0023-I-2) for WES. Exome capture and next-generation sequencing on an Illumina HiSeq2000 platform generated 50,941,902 paired-end reads of 101 nucleotides (5.1 Gigabases). Alignment to the human reference genome sequence revealed that 97.4% of targeted exonic coding regions have been covered at a minimum of 1x, 96.3% at 5x, 94.7% at 10x, 89.6% at 20x, and 86.4% at 25x.

When analyzing variants located in genes implicated in HR, including *CLCN5* [26], *DMP1* [27], *ENPP1* [28], *FGF23* [29], *SLC34A3* [30], *PHEX* [2], and *CYP27B1* [31], we found only three variants with a minor allele frequency of about 1% or below for three of those genes: *ENPP1*, *CYP27B1*, and *PHEX*. We screened the respective allele frequencies provided by “The Exome Aggregation Consortium” (ExAC; http://exac.broadinstitute.org/), which has aggregated WES data of more than 65,000 individuals worldwide. We found a heterozygous p.Val166Leu missense variant (rs8176344) in the gene *CYP27B1* with a 1.16% overall allele frequency and a specific South Asian population allele frequency of 6.78% with a PolyPhen-2 pathogenicity prediction score of 0.015 indicative for a benign polymorphism. Furthermore, we identified a heterozygous p.Leu611Val heterozygous missense variant (rs79079368) in the gene *ENPP1* with an overall allele frequency of 0.81% (2.65% in South Asian) and a PolyPhen-2 score of 0.001, also indicating the presence of a benign polymorphism. On the other hand, a heterozygous c.1080-3C>A acceptor splice site variant in intron 9 of the *PHEX* gene in the affected mother of family E0023 was absent from the 1000 Genome Project (http://www.1000genomes.org/), the ExAC database, and from the Human Gene Mutation Database (HGMD, http://www.hgmd.cf.ac.uk/ac/).

**Fig 2:**
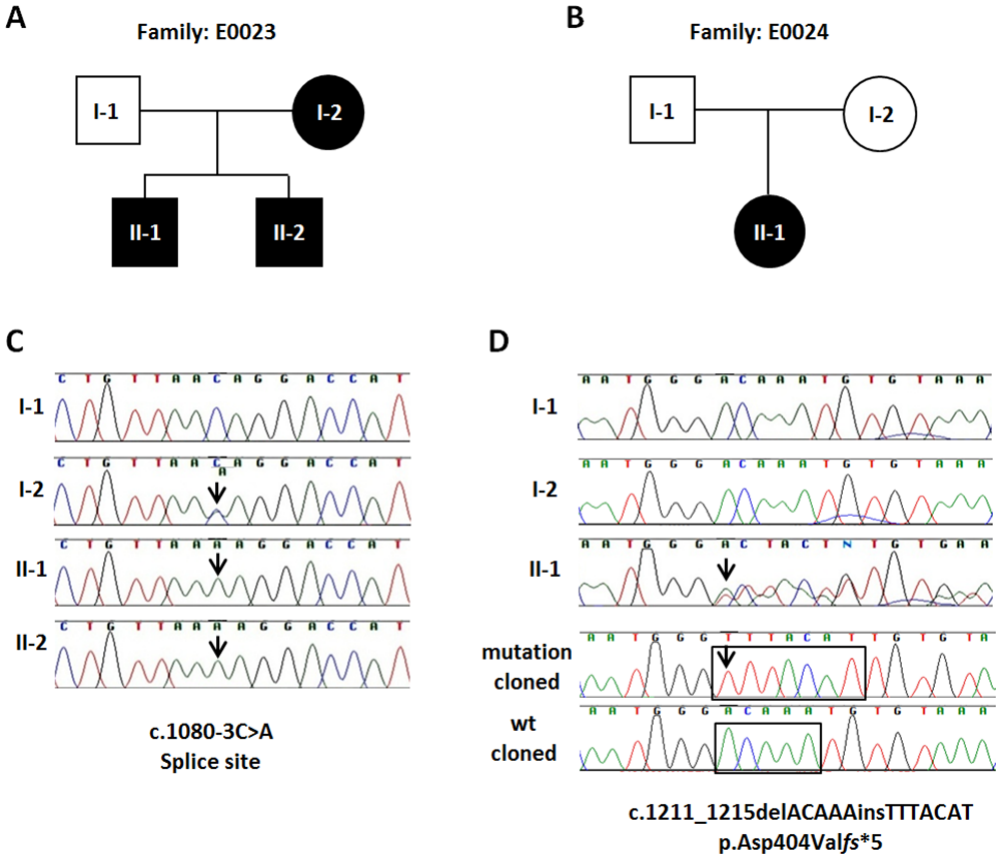
Pedigrees and *PHEX* gene mutation chromatograms from two Indian families with X-linked hypophosphatemic rickets. **(A,B)** Pedigrees of two XLH families. Filled symbols represent affected individuals. Circles and squares indicate females and males, respectively. **(C)** Genomic sequence chromatograms showing a novel acceptor splice site mutation c.1080-3C>A in intron 9 of the *PHEX* gene (arrows). The mutation in family E0023 co-segregates with the affected status and is present in the mother (I-2) and her two affected sons (II-1 and II-2). The father (I-1), who is healthy, exhibits a hemizygous wild-type allele. The mutation was identified after whole exome sequence analysis in E0023-II-2 and confirmed by direct Sanger sequencing. **(D)** A novel heterozygous insertion/deletion mutation (c.1211_1215delACAAAinsTTTACAT) in *PHEX* exon 11 leading to a frameshift (p.Asp404Valfs*5) was identified by direct Sanger sequencing in a female patient with hypohosphatemic rickets (E0024-II1). Note, that both her father (I-1) and her mother (I-2) show only the wild-type sequence. Therefore, we conclude that this mutation is most likely a *de novo* change, although the presence of germ line mosaicism in one of the parents cannot entirely be ruled out by analyzing RNA from blood only. The two bottom chromatograms represent the c.1211_1215delACAAAinsTTTACAT frameshift mutation (bottom) and the wild-type allele (above) after the respective patients’ PHEX exon 11 PCR products have been cloned into TA-cloning plasmid vector pCR2.1 (Invitrogen) and subsequently sequenced. Black boxes highlight deleted (ACAAA) and inserted (TTTACAT) bases accordingly.

Segregation analysis by direct Sanger sequencing revealed that the novel *PHEX* c.1080-3C>A variant/mutation co-segregated with the affected status and was found to be hemizygous in both affected boys. Chromatograms of the c.1080-3C>A splice site mutations are shown in Fig 2A together with the wild-type sequence derived from the healthy father.

### Genome-wide *in silico* calculation of base-level frequencies of human donor and acceptor splice sites

Splice site mutations might result in extended or skipped exons, retained introns, or the activation of cryptic splice sites. To estimate the effect of a splice site mutation we performed an *in silico* analysis and calculated the frequencies of each nucleobase at various positions around the 5’ (donor) and 3’ splice acceptor sites of all 327,293 annotated exons of the human genome.

**Fig 3:**
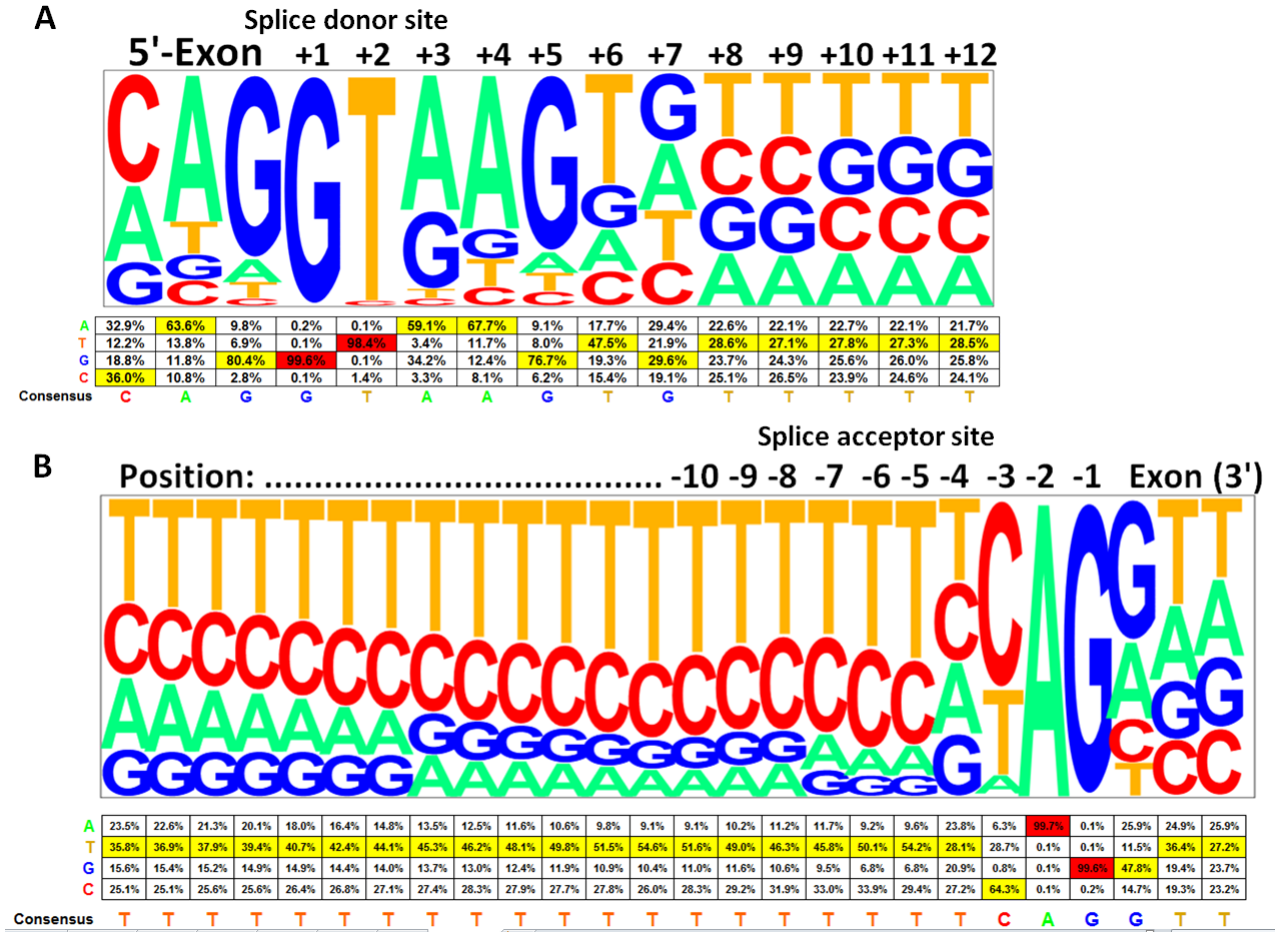
Consensus sequences and frequencies of human splice site regions. Pictograms representing the comprehensive *in silico* analysis of all human splice sites concerning 327,293 exons across 81,814 different transcripts among 20,345 human genes. **A)** Frequencies and consensus sequences of 15 human splice donor nucleobases. **B)** Frequencies and consensus sequences of 26 human splice acceptor nucleobases. Note, that the GT (+1, +2) and AG (-1, -2) positions adjacent to donor and acceptor splice junctions are highly conserved and nearly invariant. Consensus flanking bases are found at frequencies higher than expected compared to a random distribution. Frequencies of each nucleobase across various splice site positions are given in a table aligned with the respective bases in the pictograms above.

The obligatory splice sites are the nearly invariant bases of GT at the 5’ end (+1, +2) and the AG (-1,-2) at the 3’ end of the intron adjacent to donor and acceptor splice junctions. The flanking bases around the highly conserved 5’ obligatory acceptor (GT) splice site are enriched for specific nucleotides at frequencies higher than expected compared to a random distribution, especially the +3 to +6 positions with a sequence consensus of AAGT (Fig 3A). The most frequent bases upstream of the obligatory AG acceptor site are pyrimidine-rich with thymidine presenting always the most frequent base (between 28.1% and 54.6%) always followed by cytosine (between 25.1% and 64.3%) (Fig 3B). The *in silico* analysis of all human exons revealed that cytosine is the most frequent nucleobase with 64.3% at the -3 splice acceptor position, which is mutated in our patient (c.1080-3C>A), followed by thymidine with 28.7%, adenine with 6.3%, and guanine with 0.8%. We considered that the 6.3% frequency of adenine at the -3 position is indicative for a potential splice defect and performed additional RT-PCR experiments accordingly. Frequencies of each of the four possible nucleobases at each position near the 5’ and 3’ splice sites are provided in Fig 3 below the established pictograms for the splice donor and acceptor sites.

### RT-PCR

**Fig 4:**
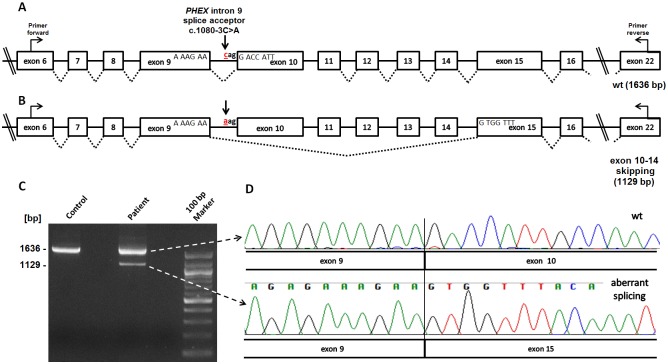
RT-PCR analysis of the mutated *PHEX* 1080-3C>A acceptor splice site. **(A)** Schematic representation of the exon/intron structure of the *PHEX* gene up and downstream of the mutated 1080-3C>A splice site in intron 9 (arrow). Primers used for amplification are indicated (PHEX_ex6_F, PHEX_ex22_R). **(B)** Schematic representation of the aberrant splice product found in male patient (E0023-II-2) who carries a hemizygous *PHEX* (c.1080-3C>A) splice acceptor site mutation (arrow). RT-PCR revealed an aberrant splice product which generates an in-frame mRNA transcript with exon 9 directly joined to exon 15, thereby skipping five consecutive exons. **(C)** Agarose gel electrophoresis of RT-PCR fragments produced after RNA was extracted from EBV-transformed peripheral lymphocytes of patient E0023-II-2 and of a healthy control individual. Using primers PHEX_exon6_forward and PHEX_exon22_reverse resulted in a 1,636 bp wild-type RT-PCR product in both samples. Note, that an additional fainter aberrant 1,129 bp splice product, which corresponds to an in-frame transcript and skipping exon 10–14, is only present in the patient’s sample. 100 bp Marker (New England Biolabs) **(D)** Sequence chromatograms of the aberrant splice product of 1,129 bp next to the sequence of the wild-type canonical splice product. Sequence traces of the aberrant fragment demonstrate that exon 9 is spliced directly to exon 15 indicating that the *PHEX* 1080-3C>A mutation may alter the strength of the intron 9 splice acceptor site.

To test whether the c.1080-3C>A *PHEX* mutation effects mRNA splicing, we performed RT-PCR experiments using RNA from immortalized B-lymphocytes derived from patient E0023-II-2 and from a healthy individual as a control (Fig 4). Immortalization of blood-derived B-lymphocytes, received from his affected brother (II-1) or his parents have been unsuccessful.

The RT-PCR amplification of *PHEX* of exon 6 to exon 22 revealed a strong 1,636 bp product when using cDNA samples as template from either the control individual or from the patient (E0023-II-2) (Fig 4). Interestingly, an additional aberrant smaller splice product of 1,129 bp was only detected in the patient (Fig 4C). Gel extraction and Sanger sequencing of the respective fainter RT-PCR product revealed that exon 9 directly splices in-frame to exon 15 and thereby skipping exons 10 to 14 (Fig 4B,4D).

### Direct Sanger sequencing of all *PHEX* exons

Direct sequencing of all *PHEX* exons and adjacent exon/intron boundaries of the affected girl (sporadic case) from family E0024 resulted in the identification of a heterozygous insertion/deletion mutation (c.1211_1215delACAAAinsTTTACAT) in exon 11. This mutation causes a frameshift at the amino acid level (p.Asp404Valfs*5) with an expected premature translation stop codon five amino acids downstream (Fig 2B). This mutation has not been reported in the HGMD database or in the locus specific (*PHEX*) mutation database (http://data.mch.mcgill.ca/phexdb). Furthermore, this mutation is absent from both the ExAC exome database, derived from 65,000 individuals, as well as from the 1000 Genomes Project’s data. Segregation analysis revealed that this novel mutation is absent from both parents’ blood DNA samples and therefore considered a *de novo* mutation.

## Discussion

In the present study, we identified two novel mutations in the *PHEX* gene in patients with HR from India. We found a novel non-obligatory splice-site mutation (c.1080-3C>A) in a family with three affected individuals and a *de novo* insertion/deletion mutation in a sporadic case with HR. Whereas the indel mutation in the sporatic case leading to a frameshift is apparent the underlying disease causing change, the pathogenicity of the splice site change found in the multiplex family (E0023), is less obvious. Thus, we used a number of bioinformatics, *in silico*, and experimental studies to support that the c.1080-3C>A mutation is indeed causing the HR phenotype in this family. This includes, i) the mutation was found in the *PHEX* gene, the most frequently gene implicated in HR, ii) no mutation was found in any other gene known to be implicated in HR when analyzing the entire WES data, iii) the mutation is absent from any public database including the ExAC data derived from 65,000 individuals of different ethnicities, iv) the mutation segregates with the affected status in the respective family with three affected members, v) nucleobase frequency analysis revealed that the minus 3 splice site change is only found in about 6% of human splice acceptor sites, vi) *in silico* tool “MutationTaster” (http://www.mutationtaster.org/) predicts that the splice site alteration is disease causing and likely disturbs normal splicing, vii), and most importantly, RT-PCR revealed an aberrant splice product which is only observed using RNA derived from lymphoblasts of a patient with the respective mutation. Although it is very likely that the c.1080-3C>A change is the underlying disease causing mutation, *in vitro* cell or *in-vivo* functional tests would provide increased confidence.

Out of 366 different mutations reported in the literature to date (HGMD) there are 65 “splicing” mutations reported. The vast majority with 52 (80%) of those splice mutations affect the almost 100% conserved obligatory consensus gt-ag (+1, +2…-2, -1) sites [32–33]. Only nine of the reported splice donor and two of the splice acceptor mutations are located outside of those canonical gt-ag sites. Interestingly, both reported uncommon acceptor mutations “c.437-3C>G” [34] and “c.850-3C>G” are located at the minus 3 position with the common cytosine changed to a guanine. Guanine at the minus 3 position is rare, present in only 0.8% of all human acceptor sites (see Fig 3). The C to A change we observed at the very same position has a substantial higher occurrence frequency of 6.3% compared to more conserved C>G changes with 0.8% across all human exons. Consequently, we decided to test the splice effect of the c.1080-3C>A mutation by applying RT-PCR and using RNA from an EBV-transformed patient’s lymphoblastoid cell line as working template. RT-PCR experiments revealed an aberrant splice transcript with five exons skipped together with an abundant wild-type transcript. Interestingly, the amount of the aberrant splice product was low compared to the correctly spliced wild-type transcript. It is known that that XLH is an X-linked dominant disease with complete penetrance because even one copy of a mutated allele causes disease in heterozygous females. Typically, affected males with hemizygous loss of function PHEX mutations do not express any wild-type transcripts, whereas females are expected to express at least about 50% of those transcripts compared to healthy individuals with two intact gene copies. The amount of wild-type transcripts in our patient with the c.1080-3C>A splice site mutation seems to be higher, perhaps in the range of even 90%, although we don’t know the expression pattern in disease relevant tissues. Even so, we speculate that already a small reduction of the unaltered full-length (wild-type) transcript may be sufficient to cause disease and that the splice mutation leads to a reduction of wild-type transcripts and consequently to an insufficient amount of wild-type protein.

## References

[1] Hanna JD, Niimi K, Chan JC. X-linked hypophosphatemia: Genetic and Clinical Correlates. Am J Dis Child. 1991;145: 865–870. 1858722 10.1001/archpedi.1991.02160080041018

[2] Holm IA, Huang X, Kunkel LM. Mutational analysis of the PEX gene in patients with X-linked hypophosphatemic rickets. Am J Hum Genet. 1997;60: 790–797. 9106524 PMC1712471

[3] Drezner MK. PHEX gene and hypophosphatemia. Kidney Int. 2000;57:9–18. 10620182 10.1046/j.1523-1755.2000.00807.x

[4] Rowe PS. 2000 The molecular background to hypophosphatemic rickets. Arch Dis Child. 2000;83: 192–194. 10952628 10.1136/adc.83.3.192 PMC1718477

[5] Tenenhouse HS, Murer H. Disorders of renal tubular phosphate transport. J Am Soc Nephrol. 2003;14: 240–248. 12506157 10.1097/01.asn.0000045045.47494.71

[6] Auricchio A, Sabbagh Y, Tenenhouse HS, Econs MJ. Mendelian hypophosphatemias In: Scriver CR, Beaudet AL, Sly WS, Valle D, Vogelstein B, eds. The Online Metabolic and Molecular Bases of Inherited Disease (OMMBID). New York, NY: McGraw-Hill Chap 197 2008 Available: http://ommbid.mhmedical.com/content.aspx?bookid=474&sectionid=45374209.

[7] Chandran M, Chng CL, Zhao Y, Bee YM, Phua LY, Clarke BL. Novel PHEX gene mutation associated with X linked hypophosphatemic rickets. Nephron Physiol. 2010;116: 17–21. 10.1159/000319318 20664300

[8] Jagtap VS, Sarathi V, Lila AR, Bandgar T, Menon P, Shah NS. Hypophosphatemic rickets. Indian J Endocrinol Metab. 2012;16: 177–182. 10.4103/2230-8210.93733 22470852 PMC3313733

[9] Cho HY, Lee BH, Kang JH, Ha IS, Cheong HI, Choi Y. A clinical and molecular genetic study of hypophosphatemic rickets in children. Pediatr Res. 2005;58: 329–333. 16055933 10.1203/01.PDR.0000169983.40758.7B

[10] Francis F, Hennig S, Korn B, Reinhardt R, Jong PD, Poustka A, et al A gene (PEX) with homologies to endopeptidases is mutated in patients with X-linked hypophosphatemic rickets. Nat Genet. 1995;11: 130–136. 7550339 10.1038/ng1095-130

[11] Dixon PH, Christie PT, Wooding C, Trump D, Grieff M, Holm I, et al Mutational analysis of PHEX gene in X-linked hypophosphatemia. J Clin Endorinol Metab. 1998;83: 3615–3623. 9768674 10.1210/jcem.83.10.5180

[12] Sabbagh Y, Boileau G, Campos M, Carmona AK, Tenenhouse HS. Structure and function of disease-causing missense mutations in the PHEX gene. J Clin Endocrinol Metab. 2003;88: 2213–2222. 12727977 10.1210/jc.2002-021809

[13] Du L, Desbarats M, Viel J, Glorieux FH, Cawthorn C, Ecarot B. cDNA cloning of the murine Pex gene implicated in X-linked hypophosphatemia and evidence for expression in bone. Genomics. 1996;36: 22–28. 8812412 10.1006/geno.1996.0421

[14] Thompson DL, Sabbagh Y, Tenenhouse HS, Roche PC, Drezner MK, Salisbury JL, et al Ontogeny of Phex/PHEX protein expression in mouse embryo and subcellular localization in osteoblasts. J Bone Miner Res. 2002;17: 311–320. 11811562 10.1359/jbmr.2002.17.2.311

[15] Guo R, Quarles LD. Cloning and sequencing of human PEX from a bone cDNA library: evidence for its developmental stage-specific regulation in osteoblasts. J Bone Miner Res. 1997;12: 1009–1017. 9199999 10.1359/jbmr.1997.12.7.1009

[16] Cheon CK, Lee HS, Kim SY, Kwak MJ, Kim GH, Yoo HW. A novel de novo mutation within PHEX gene in a young girl with hypophosphatemic rickets and review of literature. Ann Pediatr Endocrinol Metab. 2014;19: 36–41. 10.6065/apem.2014.19.1.36 24926462 PMC4049552

[17] Yue H, Yu JB, He JW, Zhang Z, Fu WZ, Zhang H, et al Identification of two novel mutations in the PHEX gene in Chinese patients with hypophosphatemic rickets/osteomalacia. PLoS One. 2014;9:e97830 10.1371/journal.pone.0097830 24836714 PMC4024000

[18] Durmaz E1, Zou M, Al-Rijjal RA, Baitei EY, Hammami S, Bircan I, et al Novel and de novo PHEX mutations in patients with hypophosphatemic rickets. Bone. 2013;52: 286–291. 10.1016/j.bone.2012.10.012 23079138

[19] Walton RJ, Bijvoet OL. Nomogram for derivation of renal threshold phosphate concentration. Lancet. 1975;2: 309–310. 50513 10.1016/s0140-6736(75)92736-1

[20] Bentley DR, Balasubramanian S, Swerdlow HP, Smith GP, Milton J, Brown CG, et al Accurate whole human genome sequencing using reversible terminator chemistry. Nature. 2008;456: 53–59. 10.1038/nature07517 18987734 PMC2581791

[21] Adzhubei IA, Schmidt S, Peshkin L, Ramensky VE, Gerasimova A, Bork P, Kondrashov AS, Sunyaev SR. A method and server for predicting damaging missense mutations. Nat Methods. 2010;7: 248–249. 10.1038/nmeth0410-248 20354512 PMC2855889

[22] Schwarz JM, Rodelsperger C, Schuelke M, Seelow D. MutationTaster evaluates disease-causing potential of sequence alterations. Nat Methods. 2010;7: 575–576. 10.1038/nmeth0810-575 20676075

[23] Kircher M, Witten DM, Jain P, O'Roak BJ, Cooper GM, Shendure J. A general framework for estimating the relative pathogenicity of human genetic variants. Nat Genet. 2014;46: 310–315. 10.1038/ng.2892 24487276 PMC3992975

[24] Otto EA, Helou J, Allen SJ, O'Toole JF, Wise EL, Ashraf S, et al Mutation analysis in nephronophthisis using a combined approach of homozygosity mapping, CEL I endonuclease cleavage, and direct sequencing. Hum Mutat. 2008;29: 418–426. 18076122 10.1002/humu.20669

[25] Chomczynski P, Sacchi N. Single-step method of RNA isolation by acid guanidinium thiocyanate-phenol-chloroform extraction. Anal Biochem. 1987;162:156–159. 2440339 10.1006/abio.1987.9999

[26] Bolino A, Devoto M, Enia G, Zoccali C, Weissenbach J, Romeo G. Genetic mapping in the Xp11.2 region of a new form of X-linked hypophosphatemic rickets. Europ J Hum Genet. 1993;1: 269–279. 7915957 10.1159/000472424

[27] Feng JQ, Ward LM, Liu S, Lu Y, Xie Y, Yuan B, et al Loss of DMP1 causes rickets and osteomalacia and identifies a role for osteocytes in mineral metabolism. Nat Genet. 2006;38: 1310–1315. 17033621 10.1038/ng1905 PMC1839871

[28] Lorenz-Depiereux B, Schnabel D, Tiosano D, Hausler G, Strom TM. Loss-of-function ENPP1 mutations cause both generalized arterial calcification of infancy and autosomal-recessive hypophosphatemic rickets. Am J Hum Genet. 2010;86: 267–272. 10.1016/j.ajhg.2010.01.006 20137773 PMC2820166

[29] Consortium ADHR. Autosomal dominant hypophosphataemic rickets is associated with mutations in FGF23. Nat Genet. 2000; 26: 345–348. 11062477 10.1038/81664

[30] Bergwitz C, Roslin NM, Tieder M, Loredo-Osti JC, Bastepe M, Abu-Zahra H, et al SLC34A3 mutations in patients with hereditary hypophosphatemic rickets with hypercalciuria predict a key role for the sodium-phosphate cotransporter NaP(i)-IIc in maintaining phosphate homeostasis. Am J Hum Genet. 2006;78: 179–192. 16358214 10.1086/499409 PMC1380228

[31] Kitanaka S, Takeyama K, Murayama A, Sato T, Okumura K, Nogami M, et al Inactivating mutations in the 25-hydroxyvitamin D3 1alpha-hydroxylase gene in patients with pseudovitamin D-deficiency rickets. N Engl J Med. 1998;338: 653–661. 9486994 10.1056/NEJM199803053381004

[32] Padgett RA, Grabowski PJ, Konarska MM, Seiler S, Sharp PA. Splicing of messenger RNA precursors. Annu Rev Biochem. 1986;55:1119–1150. 2943217 10.1146/annurev.bi.55.070186.005351

[33] Keller EB, Noon WA. Intron splicing: a conserved internal signal in introns of animal pre-mRNAs. Proc Natl Acad Sci U S A. 1984;81: 7417–7420. 6209716 10.1073/pnas.81.23.7417 PMC392157

[34] Gaucher C, Walrant-Debray O, Nguyen TM, Esterle L, Garabédian M, Jehan F. PHEX analysis in 118 pedigrees reveals new genetic clues in hypophosphatemic rickets. Hum Genet. 2009;125: 401–411. 10.1007/s00439-009-0631-z 19219621

